# Genetic Architecture of Complex Traits and Accuracy of Genomic Prediction: Coat Colour, Milk-Fat Percentage, and Type in Holstein Cattle as Contrasting Model Traits

**DOI:** 10.1371/journal.pgen.1001139

**Published:** 2010-09-23

**Authors:** Ben J. Hayes, Jennie Pryce, Amanda J. Chamberlain, Phil J. Bowman, Mike E. Goddard

**Affiliations:** 1 Biosciences Research Division, Department of Primary Industries Victoria, Melbourne, Victoria, Australia; 2 Faculty of Land and Food Resources, University of Melbourne, Melbourne, Victoria, Australia; University of Liège, Belgium

## Abstract

Prediction of genetic merit using dense SNP genotypes can be used for estimation of breeding values for selection of livestock, crops, and forage species; for prediction of disease risk; and for forensics. The accuracy of these genomic predictions depends in part on the genetic architecture of the trait, in particular number of loci affecting the trait and distribution of their effects. Here we investigate the difference among three traits in distribution of effects and the consequences for the accuracy of genomic predictions. Proportion of black coat colour in Holstein cattle was used as one model complex trait. Three loci, *KIT*, *MITF*, and a locus on chromosome 8, together explain 24% of the variation of proportion of black. However, a surprisingly large number of loci of small effect are necessary to capture the remaining variation. A second trait, fat concentration in milk, had one locus of large effect and a host of loci with very small effects. Both these distributions of effects were in contrast to that for a third trait, an index of scores for a number of aspects of cow confirmation (“overall type”), which had only loci of small effect. The differences in distribution of effects among the three traits were quantified by estimating the distribution of variance explained by chromosome segments containing 50 SNPs. This approach was taken to account for the imperfect linkage disequilibrium between the SNPs and the QTL affecting the traits. We also show that the accuracy of predicting genetic values is higher for traits with a proportion of large effects (proportion black and fat percentage) than for a trait with no loci of large effect (overall type), provided the method of analysis takes advantage of the distribution of loci effects.

## Author Summary

Prediction of future phenotypes or genetic merit using high-density SNP chips can be used for prediction of disease risk in humans, for forensics, and for selection of livestock, crops, and forage species. Key questions are how accurately these predictions can be made and on what parameters does the accuracy depend. In this paper, we use three dairy cow traits—proportion of black on coat, fat percentage in milk, and overall type, which measures cow confirmation—to demonstrate the large differences among genetic architectures of complex traits. For example 24% of the genetic variance in proportion of black is determined by three loci, *KIT*, *MITF*, and a locus on chromosome 8; however a surprisingly large number of additional loci, all of small effect, are required to capture the remaining variation. For overall type, a very large number of loci are necessary to capture the same level of variance. We also show that the accuracy of predicting genetic values is higher for traits with a proportion of large effects (proportion black and fat percentage) than for a trait with no loci of large effect (overall type), provided the method of analysis takes advantage of the distribution of loci effects.

## Introduction

Genomic prediction of future phenotypes or genetic merit using dense SNP genotypes can be used for prediction of disease risk, for forensics, and for estimation of breeding values for use in selection of livestock, crops and forage species [1]–[4]. In dairy cattle, estimated breeding values predicted from genomic information are now in wide spread use [3], [5].

The accuracy of genomic predictions will depend on the number of phenotypes used to derive the prediction equation, the heritability of the trait, the effective population size, the size of the genome, the density of markers, and the genetic architecture of the trait, in particular number of loci affecting the trait and distribution of their effects [6]–[8]. In simulated data the distribution of loci effects affects the accuracy of predicting genetic values. However in real data it has been difficult to show that traits vary in this distribution. For instance, in many cases a statistical method (Best linear unbiased prediction or BLUP) designed for traits with many loci all of small effects performs as well as methods assuming other distributions of loci effects, such as a t-distribution [9]–[10]. If it is true that most complex traits are controlled by very many polymorphisms of very small effect (a nearly infinitesimal model), this has important consequences for prediction of genetic merit or future phenotypes such as disease risk. Formulae for the accuracy of genomic prediction under this model suggest that sample sizes >100,000 individuals will be needed to achieve high accuracy, except for populations with a small effective population size [7]. Thus it is important to determine the distribution of effect sizes for a range of traits, use this information in genomic prediction and plan future experiments accordingly.

Coat colour in mammals is usually regarded as trait controlled by a few loci of large effect. However, aspects of coat colour have been suggested as a model for investigating complex trait architecture, given the close relationship between genotype and phenotype [11]. White spotting of the coat is one such “quantitative” coat colour trait, as it can be recorded as the proportion of the coat which is white. White spotting occurs in many domesticated mammals, including cattle, horses, dogs and cats. In dogs, mutations causing white spotting have been mapped to the microphthalmia-associated transcription factor (*MITF*) [12]. In mice, at least ten genes have been demonstrated to affect white spotting [13]. In horses, an inversion on chromosome 3 in the region of the Hardy-Zuckerman 4 feline sarcoma viral oncogene homolog (*KIT*) gene is associated with tobiano white-spotting pattern [14], and a seven other mutations at the *KIT* gene are associated with white coat colour phenotypes [15]. Further, mutations in *KIT* are also associated with roan coat [16]. In domestic pigs, a number of alleles of the *KIT* gene have been characterised and associations with dominant white colour demonstrated [17]. Recently, in black and white dairy cattle, variation in the degree of white spotting (measured as proportion of the coat which is black) has been mapped using linkage to large genomic intervals on chromosome 6 and chromosome 22, which contain the *KIT* and *MITF* loci respectively [18]. However a genome wide association study (GWAS) has not been reported for this trait.

Complex traits which have been studied by GWAS in dairy cattle include fat% and “type”, a complex conformation trait [19]–[20]. A single mutation in the *DGAT1* gene accounts for 30% of the variation in fat% from Holstein Friesian cattle [21]. This is in contrast to “type”, a complex trait combining scores for a number of aspects of cow confirmation (termed “overall type”), for which only modest effects have been reported.

In this paper, we use proportion of black on the coat, fat% and overall type to show that differences in the distribution of loci effects are recognisable using a new method to estimate the distribution of variance explained by each QTL. We demonstrate that three loci, *KIT*, *MITF* and a locus on chromosome 8 together explain a considerable proportion of the variation in proportion of black, but a large number of loci of small effect are necessary to capture the remaining variation. We then contrast the accuracy of genomic prediction which can be achieved for this trait with the accuracy of genomic predictions for overall type and fat% in milk. The results demonstrate a clear effect of trait architecture on the accuracy of genomic predictions.

## Results/Discussion

### Genome-wide association study for proportion of black

**Figure 1:**
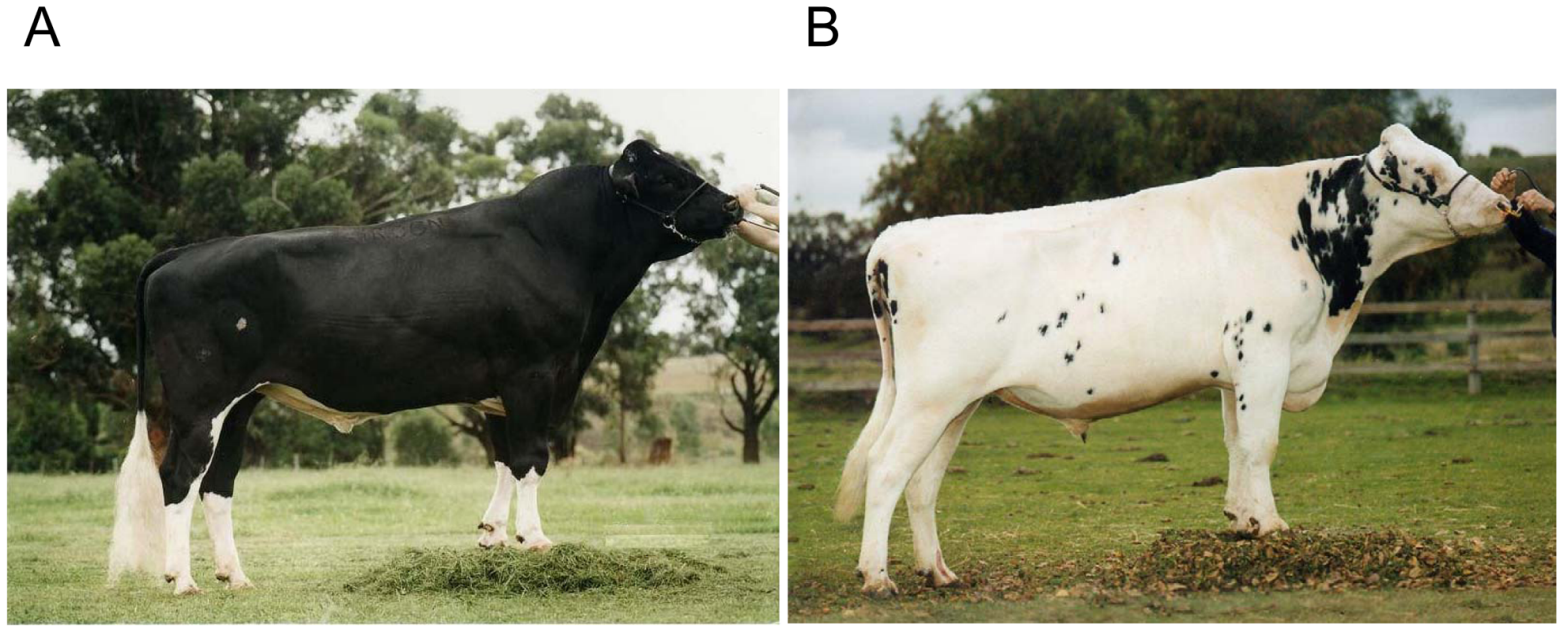
Proportion of black phenotype. Bull with 95% black (A) and bull with 5% black (B).

**Figure 2:**
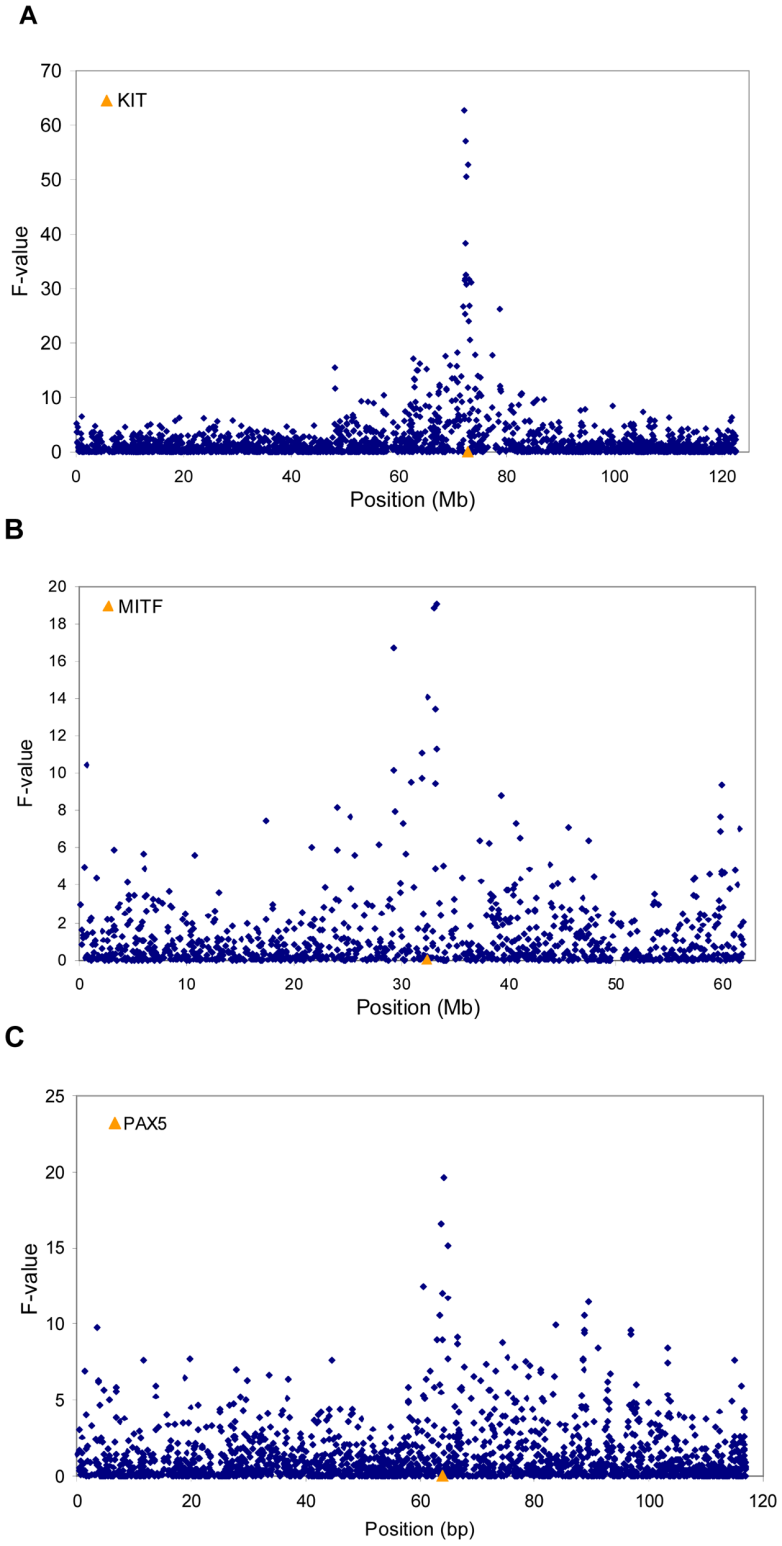
Genome-wide association study for proportion of black. Results for (A) chromosome 6, (B) chromosome 22, and (C) chromosome 8. The location of the *KIT*. *MITF* and *PAX5* genes are also indicated.

While GWAS results for fat% have been reported previously, no GWAS results have been reported for proportion of black [19]–[20]. In our population of 756 Holstein bulls, phenotypes for proportion of black varied from almost completely black to completely white, Figure 1. A GWAS study using 43115 SNPs detected three genome regions containing SNPs with P values<10^−4^ in the discovery population. We tested these in an independent validation population and confirmed three SNPs at P<0.001, Table 1 and Figure 2. The most significant SNP was within the *KIT* locus on chromosome 6 (72,104,530 bp). There was another highly significant SNP at 32,459,763 bp on chromosome 22 which is in very close proximity to the *MITF* locus (32,353,746–32,397,952 bp). There was also a highly significant SNP on chromosome 8 at 64,164,842bp. This SNP is within the zinc finger CCHC domain containing 7 gene (ZCCHC7). However zinc finger CCHC domain containing genes have not been implicated in coat colour development in any species. Perhaps a more plausible candidate in this region is *PAX5* (63,778,241–63,950,395bp). Other members of this family, *PAX3* and *PAX6*, have been demonstrated to interact with *MITF* [22]. Planque et al. [22] pointed out that the structure and docking of *PAX5* should be nearly identical to *PAX6*, because their C-terminal subdomains are 75% identical and all DNA-contacting residues are conserved [and 23]. However the interaction between *PAX5* and *MITF* remains to be demonstrated.

**Table 1:** SNPs with validated associations with proportion of black on coat in a set of 400 Holstein bulls.

SNP Name	Chrom.	Position	F-value	Effect size	Proportion of variance accounted for
ss117968126	6	72104530	76.92	14.96%	9.4%
ss86322805	8	64164842	11.40	24.97%	6.0%
ss61545343	22	32459763	32.58	10.48%	8.8%

F-value, effect size, and proportion of variance explained are all for the validation data set.

Together the three loci on chromosomes 6, 8 and 22 accounted for 24% of the variation in proportion of black phenotypes in the validation population. There was no evidence of dominance for any of these significant SNPs, and no statistical support for an interaction between the significant SNPs in *KIT*, *MITF* and the locus on chromosome 8 when we fitted models evaluating these effects.

Figure 2 also illustrates an interesting property of genome wide association studies in black and white dairy cattle, and other breeds and species with small recent effective population size. The SNP residing in the *KIT* gene has the largest F-value, exceeding the next largest SNP by 10 F units. However there are significant SNPs extending 10-15Mb either side of the most significant SNP. This is likely to be caused by the pattern of linkage disequilibrium in livestock: while at short distances levels of r^2^ between markers are similar to that observed in humans, low levels of LD (r^2^≤0.1) extend for many Mb in Holstein-Friesian cattle, probably due to recent reduction in effective population size [24].

A GWAS for fat% has been conducted in the same data [20]. Briefly, 40 SNPs had validated associations (P<0.01) for fat%, with the largest effects on chromosome 14 in close proximity to the *DGAT1* gene, and other large effects on chromosomes 2, 6 and 20. For overall type, a small number of SNPs had validated associations, however the false discovery rate in the validation population was close to 100% (Figure S1).

**Figure 3:**
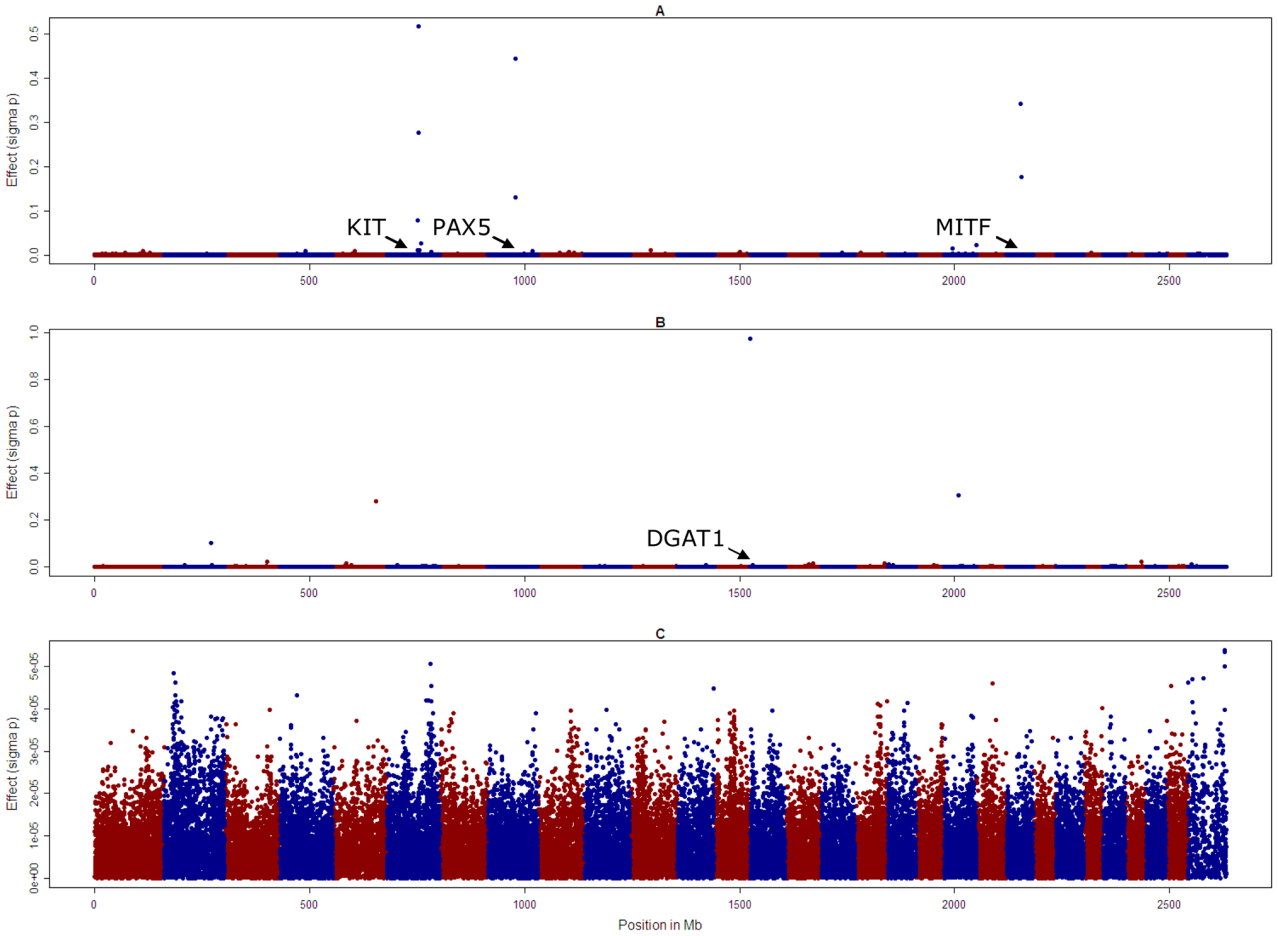
Genome-wide SNP effects when all SNPs are fitted simultaneously for three traits in Holstein Friesian cattle. Proportion of black (A), fat% (B), and overall type (C). Note the different scale of the y axis for overall type compared with proportion of black and fat%.

To overcome the tendency to find significant SNPs up to 15 Mb from a causal variant, we then used a different approach to conduct the genome wide association studies, where all SNPs were fitted simultaneously as random effects sampled from a t-distribution (method BayesA of Meuwissen et al. [25]). The effects of the SNPs associated with *KIT* and *MITF*, and the SNP on chromosome 8 that was significant in the GWAS, had the largest absolute value, but there were other smaller effects on chromosome 4, 7 and 17, Figure 3. Genome scans conducted in a similar way for fat concentration in milk (fat%) revealed large effects on chromosome 14 in close proximity to the *DGAT1* gene (443,937 bp), on chromosome 5 (position 101,015,511 bp) and 20 (34,036,832 bp) for fat%. However there were no effects greater than 5×10^−5^ phenotypic standard deviations for overall type.

### Distribution of loci effects for proportion of black, fat percentage, and overall type

Although these analyses demonstrate the importance of a small number of loci, they do not describe the complete distribution of gene effects. Estimated SNP effects will reflect both the QTL effect and the LD between the QTL and the SNP. Although the level of LD between SNP and QTL is unknown, the average level of LD (r^2^) between adjacent SNPs in our population was only 0.271. Therefore we took the approach of using chromosome segments to derive the distribution of effects as chromosome segments with multiple SNPs are more likely to capture the complete effect of the QTL. A chromosome segment was defined as consisting of 50 adjacent SNP loci. The SNPs were approximately equally spaced, such that a 50 SNP segment was 3350kb long. This size of segment was chosen as a compromise between having too little SNP information to accurately estimate its contribution to the variance, and having sufficiently small segments to enable interpretation regarding the distribution of effects on the trait. Then a genomic relationship matrix among the animals for that chromosome segment was constructed (as described in materials and methods below). To remove variance due to genes in the rest of the genome and due to population structure, a second genomic relationship matrix was constructed from all SNPs other than the 50 in the current chromosome segment. Then the proportion of variance explained by the 50 SNP chromosome segment was estimated, with both effects fitted simultaneously.

**Figure 4:**
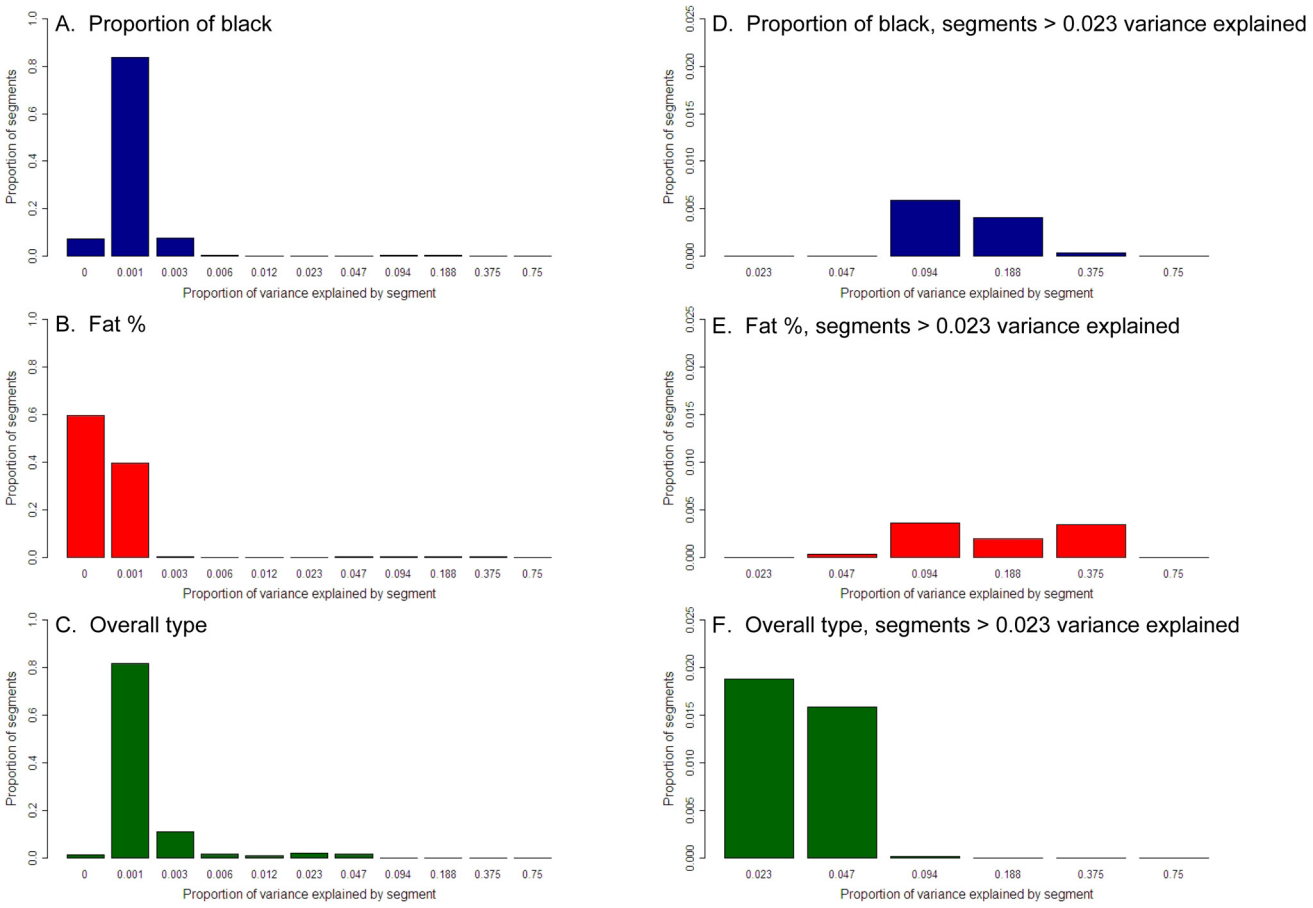
Distribution of proportion of variance explained by 50 SNP chromosome segments for three traits. Proportion of black (A), fat % (B), and overall type (C). The x axis is on a logarithmic scale. (D-F) are extreme right hand side of the same graphs, with the x axis from 0.023 to 1.0 proportion of variance explained.

However, estimates of proportion of variance explained derived in this way contain sampling error. For instance, even if a chromosome segment has no effect on the trait, the estimated variance explained can be positive (it cannot be negative because maximum likelihood estimation is restricted to the parameter space and real variances cannot be negative). This was reflected in the fact that the sum of the variances across the segments without correction for sampling error was greater than the total genetic variance. We wish to estimate the distribution of the true effects of chromosome segments rather than the distribution of estimated effects. To do this we used permutation to derive the distribution of the proportion of variance explained due to the sampling error alone. Then we used maximum likelihood to estimate the distribution of true effects (Figure 4) which, when combined with the distribution of sampling errors, would yield the observed distribution of the estimated variance explained by 50 SNP chromosome segments. When we did this, for all three traits many segments explain <0.1% of the genetic variance and for proportion black 96% of segments fall into this category. If the genetic variance contributed by the segments explaining less than 0.1% of the genetic variance is summed, such segments appear to explain half the variance for both overall type and proportion of black. However, there are tens of segments that explain 0.1–4.7% of the variance for all three traits. For proportion of black there are also a three segments explaining 4.7% to 18.8% of the variance and for fat% there are three segments explaining 4.7–37.5%. This concurs with the results of the GWAS for these traits. The total variance explained is greater then 100% because segments next to the segment containing *DGAT1*, for instance, explain a significant amount of variance, so that the variance explained by *DGAT1* is counted more than once (the total summed variances were 204%, 107%, and 213% for fat%, overall type and proportion of black).

**Figure 5:**
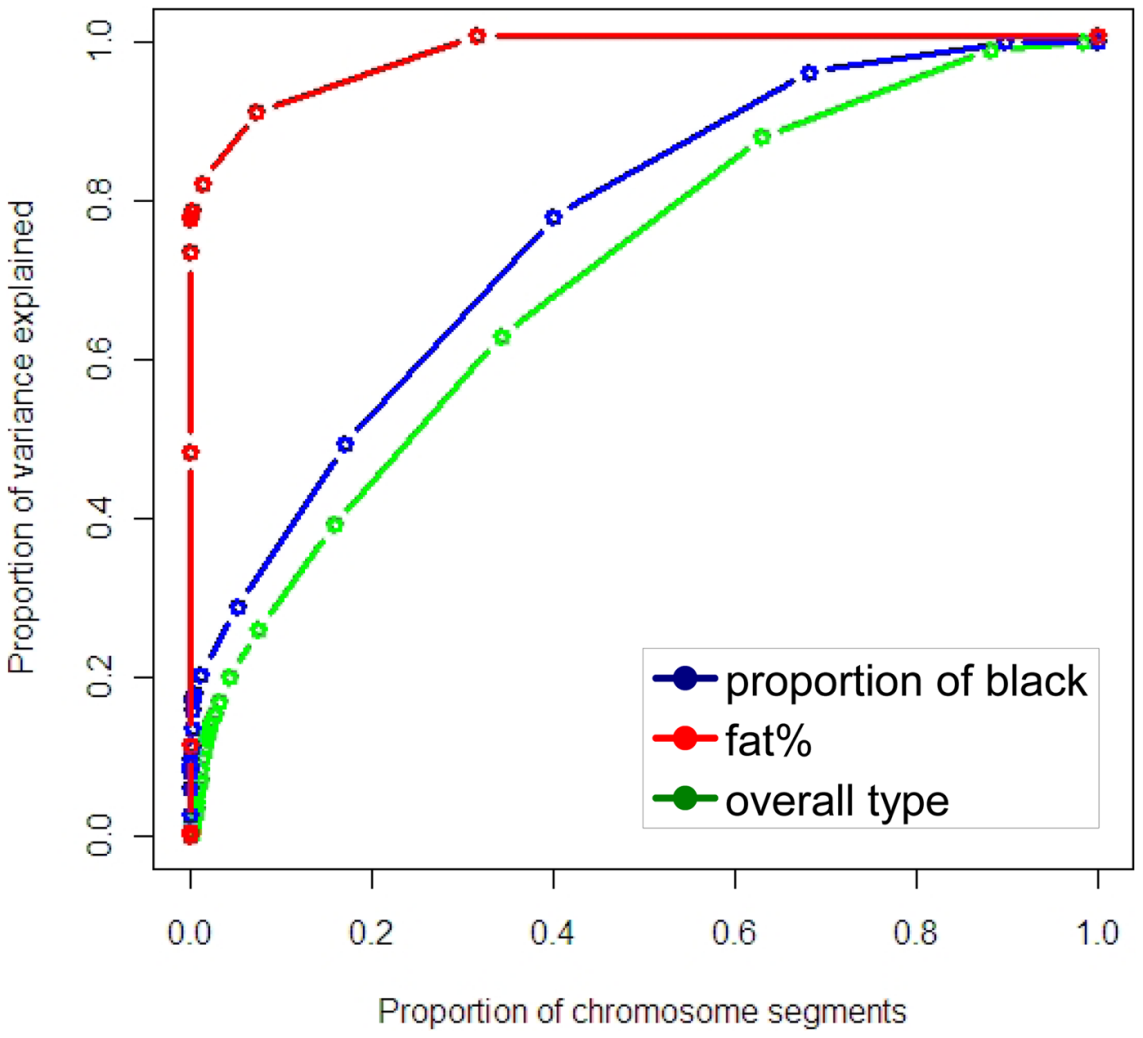
Cumulative proportion of variance explained by chromosome segments, ranked from most to least variation explained, derived from the distribution of proportion of variance explained.

The distribution of variances of chromosome segments can also be expressed as the cumulative proportion of the total variance explained when the segments are ranked from largest variance to smallest (Figure 5). The variances of the segments surrounding the segment containing *KIT*, *MITF* and the locus on chromosome 8 were set to zero so variance caused by these mutations was not double counted. The same procedure was used for the segments surrounding the *DGAT1* gene and other large effects for fat%. For proportion of black and particularly fat%, a small proportion of segments are necessary to capture a significant proportion of the variance, while for overall type a greater number of segments are required. Note that the sum of the variance from the segments explaining the largest proportion of the variance is now 20%, compared with 24% estimated from the GWAS. This reflects the fact that the estimate of variance explained is regressed to account for estimation error. For proportion of black, as segments other than the three containing *KIT*, *MITF* and the significant SNP on chromosome 8 only explain a small proportion of the variance, many of them are required to explain even the majority of the variance.

### Effect of the distributions of loci effects on the accuracy of genomic prediction

To investigate the effect of the distributions of loci effects on the accuracy of genomic estimated breeding values, we used SNP effects for each trait from the Bayesian approach described above to predict genomic estimated breeding values. This was done for the independent validation population of 400 bulls, as 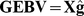, where **X** is a matrix with a row for each animal and a column for each SNP and X_ij_ is the number of “2” alleles where they alleles are designated 1 or 2, 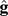 is a vector containing the estimate of the size of the effect of marker (the effect of inheriting on copy of allele 2) when the effect of the first allele is set to zero. The phenotypes of the animals in the validation population were not used to predict the SNP effects. To estimate the accuracy of the GEBV we used the correlation between it and the phenotype of each animal corrected for the correlation of the phenotype with the true genetic value. The accuracies of genomic estimated breeding value were 0.56, 0.69 and 0.80 for overall type, proportion of black and fat% respectively, Table 2. The accuracy of these GEBVs was compared to that obtained using a statistical analysis (BLUP) that assumed all SNP effects are sampled from a normal distribution and therefore no large effects exist. These accuracies of the GEBVs using the Bayes A method were higher than those using the BLUP method for fat% and proportion of black but lower for overall type, Table 2.

**Table 2:** Deterministic predictions of accuracy of genomic breeding value (GEBV) and realised accuracies of genomic breeding values.

	*Trait*		
	Overall type	Proportion of black	Fat %
Number of records in reference set	756	327	756
Heritability of records	0.63	0.74	0.83
Deterministic prediction of accuracy of GEBV			
Normal distribution of effects	0.35	0.26	0.39
Leptokurtotic distribution of effects	0.75	0.66	0.93
Realised accuracy of GEBV			
BLUP	0.42	0.46	0.63
BAYESA	0.38	0.59	0.73

The deterministic predictions assumed either normal or leptokurtotic (t, degrees of freedom = 4.012) distributions of effects, a genome length of 30 Morgans and effective population size of 100 (Goddard 2008, as modified by Hayes et al. 2009).

**Figure 6:**
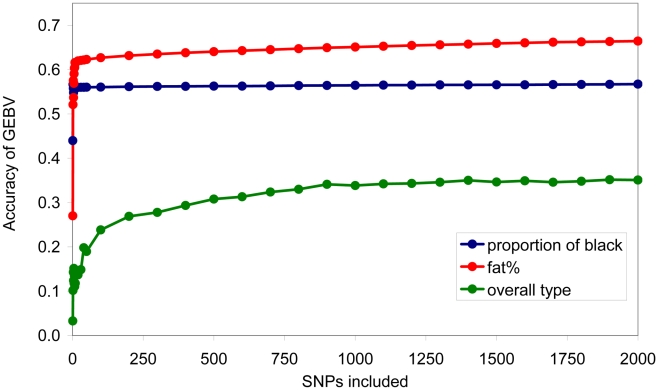
Accuracy of genomic breeding value (GEBV) when a given number of SNPs of largest effect are used to calculate the GEBV.

GEBV was also calculated using subsets of SNPs ranked in order of the size of their effect. For each subset, BayesA was re-run to predict SNP effects. For proportion of black, a very small number of SNPs were required to achieve close to 95% of the accuracy possible with the full set of SNPs, while at the other extreme for overall type 2000 SNPs were required to achieve greater than 90% of the accuracy possible with the full set of SNPs, Figure 6.

For traits with a few moderate effects, and many small effects, such as proportion of black, the accuracy of estimating the moderate effects will be much higher than the accuracy of estimating the very small effects. There is also a large effect of the number of records used to estimate the effects – for proportion of black there were only 327 records while for the other traits there were 756 records. When the estimated effects are used in a prediction equation for estimated breeding values, the moderate effects therefore contribute the overwhelming majority of the total accuracy of prediction. With a small number of phenotypic records, the estimates of segments with small effects can be so inaccurate that they contribute nothing to the accuracy of prediction. This explains the apparent discrepancy between Figure 5, where many chromosome segments are needed to capture the total genetic variance of the trait, and Figure 6, where close to the maximum accuracy of prediction achievable with all SNPs (0.59, Table 2) is achieved with less than 10 SNPs.

### Effect of architecture of complex traits on genomic predictions

Our results demonstrate that large differences exist in the architecture of different complex traits. For both proportion of black and fat% there are segregating mutations of moderate effect so that the distribution of effects is leptokurtotic. This in contrast to overall type which has only loci of small effect, and the distribution of these effects could be assumed to be normal.

Information on the degree of leptokurtosis of the distribution of effects can be used to guide the design of experiments that will subsequently enable genomic predictions. A deterministic method has been developed to predict the accuracy of genomic estimated breeding values [7]. The parameters of this formula were the number of phenotypic records in the reference population (N), the heritability of the trait (h^2^), the length of the genome (L), and the distribution of QTL effects. The distribution of effects could be either normal or leptokurtotic. When a normal distribution of effects is assumed, the accuracy of genomic breeding values can be predicted as 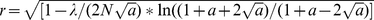 where *a* = 1+2 λ/*N*, and *λ = qk/h^2^*, with *k = 1/log(2N_e_)*, where *N_e_* is the effective population size. The parameter *q* = number of independent chromosome segments in the population. The value of *q* used here was *2N_e_L*, where *L* is the length of the genome in Morgans. Using the same number of phenotypic records as were used in our experiment, and the same heritabilities of the traits, the deterministic prediction of accuracies are given Table 2. For leptokurtotic distributions, there is no closed form equation for the accuracy of breeding values, but these accuracies can be derived by numerical integration of the accuracy of predicting the effects given the assumed distribution and allele frequencies [7]. A *t* distribution with 4.012 degrees of freedom was used to model the distribution of effects, and a U shaped distribution of allele frequencies as expected under the neutral model was used [25]. As expected, the leptokurtotic distribution of effects gave higher predicted accuracies of genomic breeding value than a normal distribution of effects. The observed accuracy of GEBVs for overall type in our experiment, 0.35, matches closely the prediction for accuracy of GEBV for a quantitative trait with the same heritability and a normal distribution of effects. Conversely, both fat% and proportion of black better match the predictions when a leptokurtotic distribution of effects was used.

The maximum accuracy for GEBVs should be obtained when the assumed distribution of effects matches the true distribution [7]. In the absence of knowledge about the true distribution two extreme approaches have been used. In one all SNP effects are assumed to come from a single normal distribution (the analysis called BLUP above). In the other only a small number of highly significant and validated SNPs from GWAS are used. For example, vanHoek et al. [26] used 9 validated genetic polymorphisms to predict disease risk for type 2 diabetes. In their study, the value of the SNP information was low, with only marginal improvement as a result of using the genetic polymorphisms beyond clinical characteristics. In this paper we have demonstrated that for some traits, such as overall type, a large number of SNPs will be required to predict the trait with any accuracy. An approach where all SNPs are fitted simultaneously to derive a prediction equation, ignoring significance levels, should lead to higher accuracies of prediction, than an approach which uses only associations detected in GWAS with stringent thresholds. The accuracies achievable with this approach can be predicted deterministically provided we have some knowledge of whether the distribution of QTL effects is normal or leptokurtotic. The deterministic results agree only reasonably well with those we observed for proportion of black, fat% and overall type, suggesting that further knowledge about the distribution of effect would be beneficial. However, even with current knowledge the deterministic approach can be used to design experiments to develop genomic predictions.

It interesting to speculate on why large effects are segregating for fat% and proportion of black, but not overall type. For fat%, the fact that *DGAT1* continues to segregate in the population may reflect the change in breeding goal for dairy cattle over time [21]. The mutant allele decreases milk fat yield but increases milk volume so artificial selection is likely to have favoured it at times but not consistently. This swept the allele to moderate frequencies in the population. Mutations causing white spotting must have been selected by breeders of black and white cattle since it is their defining feature. Thus in both cases, mutations which would have been unfavourable before domestication, were selected and still segregate at intermediate frequencies. Overall type has also been subject to artificial selection pressure since domestication. However, any mutations of large effect would have a detrimental effect on overall fitness (natural and artificial) and would likely have been quickly removed from the population.

There is little evidence for alleles of large effect for most complex traits [27]. Thus most complex traits are like overall type in architecture. Fat% and proportion black may be examples of transient situations where a change in selection pressure has driven a mutation to intermediate frequency. Recently Eyre-Walker [28] argued that rare alleles of large effect should explain much of the variation in complex traits if there is natural selection for the trait. Our results suggest that if alleles of large effect do exist, they are at such low frequency that they individually explain a small proportion of the variance. For overall type and proportion of black at least we find that the majority of variance is contributed by a large number of chromosome segments, each explaining a small proportion of the total variance. The question is then do the segments explain a small proportion of the total variance because they harbour QTL of small effect at moderate frequency, or because they harbour QTL of large effect at very low frequency. While our experiment cannot answer this question directly, some evidence that the former explanation might be true comes from linkage experiments. Linkage experiments can estimate QTL effect sizes directly, rather than through SNP in LD with the QTL, as the association of the marker and QTL within families will be almost perfect, provided enough markers are used. Provided at least one sire in the experiment is heterozygous at the QTL, a QTL of large effect should be detected. However, despite quite large linkage mapping studies in dairy cattle with many sires and very large numbers of progeny, very few QTL of large effect were found for complex traits [29]–[30]. One exception was the DGAT1 region on chromosome 14, which was highly significant in many linkage mapping experiments [eg 21]. Taken together, our results and the results of the linkage mapping studies suggest that, although mutations of moderate effect occur (as demonstrated here for fat% and proportion black), they are very rare for complex traits compared to mutations of small effect. Our results have some implications for explaining the “missing heritability” in GWAS of human population data [27]; namely that some of the missing heritability is explained by mutations with very small effects on the trait (undetectable by GWAS), but there are very many of them. Dairy cattle have some advantages for studying this question because large amounts of data are available through the breeding programme, because analyses of sires with large numbers of tested progeny produce traits with high effective heritabilities and because the LD structure may be relatively favourable for capturing genetic variance with 10-fold fewer markers than are used in humans. However it must be pointed out that conclusions results from cattle may not be relevant for other species: the larger LD blocks in cattle than other species will mean more variance per “effective” locus than in populations with larger effective population size. Further, the history of cattle domestication with at least two separate domestications followed by hybridisation events and strong artificial selection may produce unusual patterns of diversity and LD and the distribution of allele effects may owe more to recent population demographics and artificial selection than to the natural selection for fitness that will drive other populations including humans.

## Materials and Methods

### Samples and SNPs

The data set consisted of 1200 Australian Holstein bulls. For fat% and overall type the ‘phenotype’ used for each bull was the mean phenotype of his daughters. To obtain this phenotype we de-regressed the Australian breeding values (ABVs) to remove the contribution from relatives other than daughters [3] while retaining the correction for non-genetic effects such as herd. All bulls with de-regressed estimated breeding values had at least 80 daughters. The traits measured in the bull's daughters were fat% in a sample of the milk on each test day, and overall type. Overall type is composite trait combining scores for a number of aspects of the cow's conformation, including frame-capacity, rump, feet and legs, fore udder, rear udder, mammary system and dairy character (see http://www.adhis.com.au/ for more details). For portion of black, each bull himself was scored according to the proportion of black on the entire body, from 0% to 100% black. The values ranged from 5% black to 95% black. The scorer was the same for all the bulls.

The bulls were genotyped for the Illumina Bovine50K array, which includes 54,001 Single Nucleotide Polymorphism (SNP) markers [31]. The following criteria and checks were applied to the bull's genotypes. Mendelian consistency checks revealed a small number of either sons who were discordant with their sires at many (>1000) SNPs or sires with many discordant sons. These animals (17) were removed from the data set. We omitted bulls if they had more than 20% of missing genotypes. 1181 bulls passed these criteria.

Criteria for selecting SNPs were; less than 5% pedigree discordants (eg. cases where a sire was homozygous for one allele and progeny were homozygous for the other allele), 90% call rate, MAF>2%, Hardy Weinberg P<0.00001. 40077 SNPs met all of these criteria. A small number of these were not assigned to any chromosome on Bovine Genome Build 4.0, and were omitted from the final data set, as were SNPs on the X chromosome. Parentage checking was then performed again, and any genotypes incompatible with pedigree were set to missing.

To impute missing genotypes, the SNPs were ordered by chromosome position. All SNPs which could not be mapped or were on the X chromosome were excluded from the final data set, leaving 39,048 SNPs. To impute missing genotypes, the genotype calls and missing genotype information was submitted to fastPHASE chromosome by chromosome [32]. The genotypes were taken as those filled in by fastPHASE. The accuracy of imputing genotypes was 98.6% [5].

The discovery dataset consisted of bulls progeny tested before 2004 (n = 756). For proportion of black portion 327 bulls in the reference set had phenotypes. The bulls in the validation dataset were progeny tested during or after 2004 (n = 400)

### Genome-wide association study for proportion of black

In the discovery set of bulls, a linear model was fitted to the bull's proportion of black phenotypes to determine if the SNPs accounted for any variation. The top–bottom called genotypes were re-coded as 0 for the homozygote of the first alphabetical allele, 1 for the heterozygote, and 2 for the homozygote of the second alphabetical allele. The effect of each SNP was estimated in turn using the model 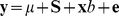 where **y** is a vector of proportion of black, *μ* is the mean, **S** is the (random) effect of the sire of each bull, **x** is a vector of genotypes, b is the effect of the SNP, and e is a vector of random residuals. The variance of the sire effects was **I**σ^2^_S_ where **I** is an identity matrix and σ^2^_S_ is the sire variance. Fitting the sire effect should remove any spurious associations due to family structure. All data analyses were performed using mixed linear models with variance components estimated by residual maximum likelihood [33]. SNPs that were significant at P<0.0001 were fitted in the validation set using the same model as above.

### Genomic prediction

#### Best linear unbiased prediction (BLUP)

If there are many QTLs whose effects are normally distributed with constant variance, then genomic selection is equivalent to replacing the expected relationship matrix with the realised or genomic relationship matrix (**G**) estimated from DNA markers in the BLUP equations. [7], [34]–[37]. The model was
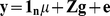
Where **y** is a vector of phenotypes, μ is the mean, **1_n_** is a vector of 1s, **Z** is a design matrix allocating records to estimated breeding values, **g** is a vector of breeding values and **e** is a vector of random normal deviates ∼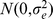. The breeding value **g** can be modelled by the combined effects of all the SNPs **g** = **Wu** where *u_j_* is the effect of the *j^th^* SNP, and 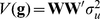. Elements of matrix **W** are *w_ij_* for the *i^th^* animal and *j^th^* SNP, where *w_ij_* = 0−2*p_j_* if the animal is homozygous 11 at the *j^th^* SNP, 1−2*p_j_* if the animal is heterozygous and 2−2*p_j_* if the animal is homozygous 22 at the *j^th^* SNP, where *p_j_* is the allele frequency of the 1 allele of the SNP. The diagonal elements of **WW′** will be 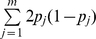 where *m* is the number of SNPs. If **WW′** is scaled such that 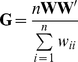 then 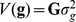.

Estimated breeding values for both phenotyped and non-phenotyped individuals can be predicted by:
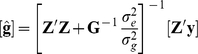
Where **G** is the realised relationship matrix calculated as above, and 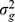 is a genetic variance. Variance components were estimated with ASREML [33].

The realised accuracy of GEBV was calculated as r(**GEBV,y_val_**)/*h* where y_val_ was the phenotype (either deregressed estimated breeding values for overall type and fat%, or the bull's own proportion of black), for bulls in the validation set, and *h* is the correlation between the phenotype and the true breeding value estimated, (the square root of the heritability of the records was used).

#### BayesA

A Bayesian approach to simultaneously predicting the effect of all SNPs to derive the prediction equation was used, namely BayesA [25]. BayesA has a prior assumption that SNP effects are t-distributed. The model fitted was:
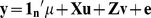
Where **y** is a vector of *n* phenotypes, **X** is (*n*×*m*) a design matrix allocating records to the marker effects with element X_ij_ = 0, 1 or 2 if the genotype of animal *i* at SNP *j* is 11, 12 or 22 respectively. **u** is a (*m*×*1*) vector of SNP effects assumed normally distributed 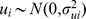, **e** is a vector of random deviates where 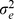 is the error variance, *v_i_* is the polygenic breeding value of the *i^th^* animal, with variance 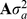, where **A** is the numerator relationship matrix derived from pedigrees. In BayesA the prior for 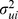 was an inverse chi square distribution with 4.012 degrees of freedom. This describes a moderately leptokurtotic distribution [25]. Using the predicted SNP effects from each method, we predicted the GEBVs in the validation sets as 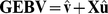. The realised accuracy of GEBV was derived as described for BLUP above.

### Estimating the distribution of the proportion of variance explained by chromosome segments

For each 50SNP segment of chromosome, we estimated the proportion of variance explained by building a genomic relationship matrix (as described above) based on the 50SNPs only (**G_1_**), and a second genomic relationship matrix (**G_2_**) using all SNPs except those in the current 50 SNP segment. We the fitted the model
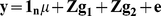
Where **y** is a vector of phenotypes, μ is the mean, **1_n_** is a vector of 1s, **Z** is a design matrix allocating records to animals, **g_1_** is a vector of genetic effects for a 50 SNP segment, assumed to be normally distributed with mean 0 and co(variance) 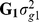, **g_2_** is a vector of breeding values based on all the other segments, assumed to be normally distributed with mean 0 and co(variance) 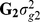 and **e** is a vector of random normal deviates ∼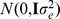. Variance components were estimated with ASREML [33], and the proportion of variance explained by each segment was calculated as 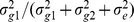.

The estimate of the proportion of variance explained by a chromosome segment *i* (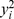) is naturally subject to some sampling error. 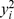 is analogous to the squared correlation between the effect of the segment and the phenotype so *y_i_* is analogous to the correlation. We modelled y_i_ as 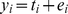 where *t_i_* is the true correlation between segment *i* and phenotype and *e_i_* is a sampling error While it is not possible to estimate the sampling error for a specific segment, we can estimate the distribution of sampling errors. To do this the phenotypes were permuted across the genotypes 1000 times and the proportion of variance explained by each segment re-calculated. Under the null hypothesis that there is no real correlation between segments and phenotypes, the distribution of the estimated proportion variance explained should be a mixture of zero and a chi-square with 1 degree of freedom (half the time the correlation would be estimated to be negative but maximum likelihood always reports an estimate within the parameter space and so half he reported estimates of variance are zero). Therefore the square roots of these estimates were assumed to be near-zero (half the time) and the positive half of a normal distribution the other half. The standard deviation of e, σ, was then taken as the square root of the average proportion of variance explained multiplied by 2 (the multiplication by two was to account for the fact that negative estimates of the proportion of variances explained are reported as zero).

We then used maximum likelihood to estimate the distribution of true chromosome segment variances (*t_i_^2^*) given that we had a sample of estimated chromosome segment variances (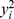) and 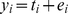 with *e_i_*∼N(0,σ).

We estimate the distribution of *t* and then convert that to a distribution of *t^2^*. We did not wish to assume any parametric form for the distribution of t so we approximate it by a discrete distribution in which the proportion explained can only take values *j* = 0.00, 0.005 and so on to 1 (eg 100 classes between 0 and 1, but including 0). We then estimate the frequency of these discrete values. The probability of observing 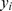 given *j* and *σ* was taken as 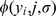 if 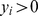 and 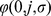 if 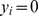 where 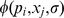 is the density function of the normal distribution and 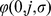 is the cumulative function of the normal distribution. (If *t+e* is negative for a segment then *y^2^* would be reported as zero since negative variances are not allowed).

Then an expectation maximisation (EM) algorithm was used to estimate the proportion of chromosome segments in each class *f_j_*. The EM algorithm had three steps

1. Initialise each *f_j_*. to 0.01.
2. Calculate the probability of the j given the y_i_ was 
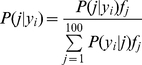

3. Update the proportion of chromosome segments in each class as 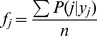

Steps 2 and 3 were repeated until the *f_j_* values did not change between iterations. The results (Figure 4) are presented as a distribution of *t^2^* where the frequencies all values of *t* between √0.01 and √0.03 are summed and presented as the frequency of 0.01<*t^2^*<0.03 etc.

## Supporting Information

Figure S1 Genome-wide association study for overall type, by chromosome and position. Light grey dots are significance levels in the Holstein discovery population, black dots are SNPs which significant in the discovery population at P<0.0001 and were also significant in the validation population at P<0.05.
(0.03 MB PDF)
