# Supplementary figures and images for: Genetic Architecture of Complex Traits and Accuracy of Genomic Prediction: Coat Colour, Milk-Fat Percentage, and Type in Holstein Cattle as Contrasting Model Traits
Source: PLoS Genet. 2010 Sep 23;6(9):e1001139. doi: 10.1371/journal.pgen.1001139 (PMC2944788; doi:10.1371/journal.pgen.1001139)

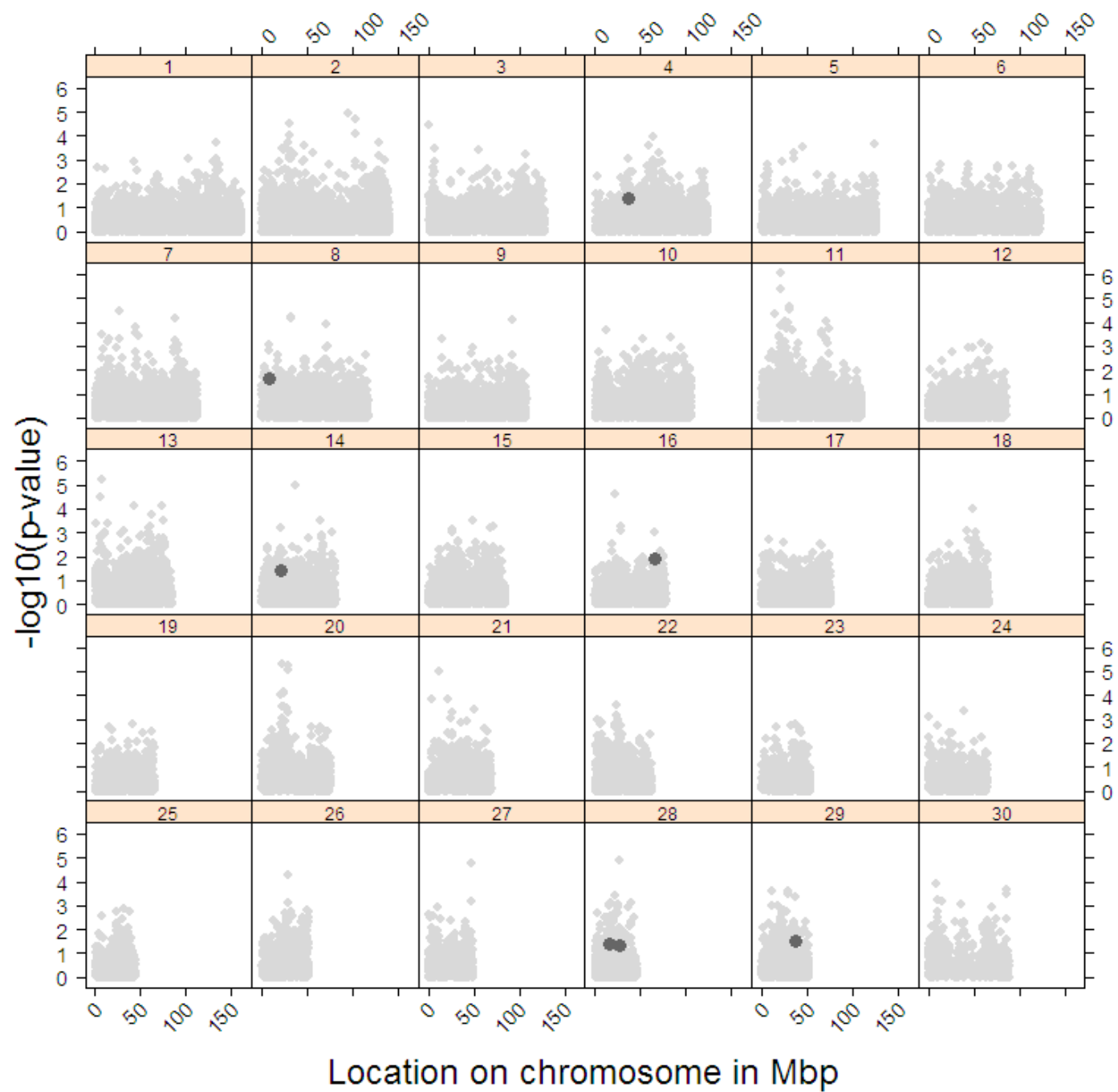

Supplement: Figure S1 — Genome-wide association study for overall type, by chromosome and position. Light grey dots are significance levels in the Holstein discovery population, black dots are SNPs which significant in the discovery population at P<0.0001 and were also significant in the validation population at P<0.05. (0.03 MB PDF) [file pgen.1001139.s001.pdf]
